# Surgical Cases

**Published:** 1889-12-21

**Authors:** 


### SURGICAL CASES.

A malignant nasal polypus was removed by splitting the nose. A woman, aged 68, began to suffer from repeated attacks of epistaxis from the right nostril; she had also noticed some offensive discharge. There was swelling of the right cheek and right nostril, and its cavity was blocked by a dark fleshy mass. The nose was split by passing a sharp-pointed bistoury into the right nostril, perforating the soft tissues close to the junction of the nasal bone cartilage and septum, and then cutting outwards to the tip. The growth was removed in pieces until its attachment could be seen, which was to the mucous membrane over the middle turbinated bone. The cavity was dusted with iodoform, and the wound carefully sewn up with carbolised silk. Mr. Haslam remarks, that for this method of opening a nose, he is indebted to Mr. Furneaux Jordan. It answered the purpose admirably. The complete removal of nasal polypus is often difficult, and frequent recurrences are common, but by this operation the attachment may be seen and the polypus may be removed in an effective manner. There is an objection, however, to the operation, as there is the risk of a scar in a prominent position; but by careful sewing up, the vascular tissues of the nose unite without any appreciable disfigurement. If we mistake not, this operation will eventually be preferred instead of the more uncertain process of evulsion. Mr. Haslam has not, however, given us sufficient reason why he styles this case "malignant polypus."

